# ABC transporters knockout *in Aedes aegypti* induces upregulation of paralogous genes, avoiding resistance development to *Bacillus thuringiensis* Cry toxins

**DOI:** 10.1371/journal.pone.0327221

**Published:** 2025-07-03

**Authors:** Sabino Pacheco, Marcos Chiñas, Juan Ulises Gómez, Ángel Enrique Peláez-Aguilar, Nathaly Alexandre do Nascimento, Pablo Emiliano Cantón, Jorge Sánchez, Samira López-Molina, Isabel Gómez, Mario Soberón, Alejandra Bravo

**Affiliations:** Departamento de Microbiología Molecular, Instituto de Biotecnología, Universidad Nacional Autónoma de México (UNAM), Cuernavaca, Morelos, Mexico; PMAS Arid Agriculture University: PMAS-Arid Agriculture University Rawalpindi, PAKISTAN

## Abstract

ABC transporters are membrane proteins that modulate the insecticidal activity of *Bacillus thuringiensis* Cry toxins by acting as receptors in the gut epithelium of insect larvae. However, their role as Cry receptors in dipteran species remains unknown. Here, we identified the ABC transporter orthologs in the *Aedes aegypti* genome corresponding to the Cry toxin receptors described in lepidopteran and coleopteran species. Analysis of their transcriptomic regulation in gut tissue, revealed the expression of ABCA2, ABCB1, and ABCC2. Using CRISPR-Cas9, we knocked out (KO) the expression of these three *ABC* transporter genes and performed *in vivo* binding assays and bioassays with four mosquitocidal Cry toxins (Cry4Aa, Cry4Ba, Cry11Aa, and Cry11Ba). Histopathological analysis showed a significant reduction of Cry toxins binding to the apical microvilli membrane of gut epithelium, depending on the ABC KO mutation. However, similar larvicidal activity was observed for Cry toxins between ABC KO and wild type strains. Transcriptomic analysis revealed specific upregulation of their corresponding ABC transporter paralogous, suggesting a compensatory mechanism that could mitigate the fitness cost of the ABC KO and potentially contribute to redundant receptor function of Cry toxins. These findings provide evidence that ABC transporters may act as physiological receptors for insecticidal Cry toxins in dipteran insects.

## Introduction

*Bacillus thuringiensis* (Bt) produces crystal-shaped inclusions during sporulation formed by insecticidal proteins such as Cry toxins. These toxins are widely used as biopesticides to control agricultural insect pests, thus reducing the need for chemical pesticides [1]. Bt subsp. *israelensis* (Bti) produce Cry proteins that are highly toxic to dipteran insect species such as *Aedes*, *Culex* and *Anopheles*. Consequently, Bti is used globally as biolarvicide to control vector-borne diseases including dengue, Zika, chikungunya, West Nile fever and malaria [2].

Larvae stages are particularly susceptible to Cry toxins, once the larvae ingest the crystal inclusion, it is solubilized in the gut lumen and Cry protoxins are activated by trypsin-like proteases. It was proposed that both protoxin or activated toxin bind to protein receptors located on the apical microvilli of the epithelial cells, triggering assembly of an oligomeric Cry structure or pre-pore. Afterward, the pre-pore is inserted into the membrane forming a pore that breakdown the cell integrity [3,4]. In *A. aegypti* larvae, it was observed that Cry toxins are distributed through the gut microvilli but are enriched in the hindgut and caeca region [5]. The proposed mode of action of Cry toxins included several membrane proteins that might function as Cry toxin receptors, such as cadherin-like proteins (Cad), aminopeptidases-N (APN) and membrane-bound alkaline phosphatases (mALP) [6–8]. However, it was recently reported that CRISPR-Cas9 knockout (KO) of these three proteins did not change the susceptibility of *A. aegypti* to Cry toxins [9,10]. Prohibitin (PHB) protein was also identified as putative receptor for Cry4Ba toxin in the CCL-125 epithelial cell line derived from *A. aegypti* mosquito, but whether it functions as a receptor in the insect larvae remains unknown [11].

Some members of the membrane ATP-binding cassette (ABC) transporters superfamily have also been described as Cry toxin receptors. In Lepidoptera, mutations in ABCC2 transporter have been associated with high levels of resistance to Cry1A toxins in insect pest such as *Heliothis virescens*, *Trichoplusia ni* and *Plutella xylostella* [12,13]. The resistance phenotype linked to ABCC2 was functionally confirmed by CRISPR-Cas9 gene editing that, nevertheless, showed lower resistance levels compared to resistant insects linked to natural ABCC2 mutations [14,15]. It was also shown that the paralogous ABCC3 was also involved in Cry toxin activity, since ABCC3 KO decreased insect susceptibility to Cry proteins, although the insect resistance was lower in comparison to ABCC2 KO. Interestingly, the simultaneous double KO of ABCC2 and ABCC3 (ABC2/C3 KO) resulted in higher resistance than the corresponding single KO, suggesting a redundant role of these proteins as receptors for Cry toxins [14–17]. Moreover, down-regulated expression of ABCG1 in *Ostrinia furnicalis* and *P. xylostella* confers resistance to Cry1A toxins, indicating that this protein also serves as receptor [18,19]. On the other hand, it has been reported that mutations in ABCA2 transporter are linked to resistance to Cry2A toxins in *Helicoverpa armigera*, *Helicoverpa punctigera* and *Pectinophora gossypiella* [20,21]. In coleopteran, a genetic analysis of a *Chrysomela tremuela* strain resistant to Cry3Aa showed that a deletion of 4 bases at ABCB1 ORF is linked to high levels of Cry3Aa resistance [22]. Its orthologous protein in another coleopteran insect, *Diabrotica virgifera virgifera*, was also identified as receptor for Cry3A toxin [23].

The ABC transporters are distributed in all organisms, and they are involved in the active transport of diverse molecules through the cell membrane. In dipteran, members of ABC transporter are associated with pyrethroid resistance, suggesting their involvement in the detoxification of xenobiotic compounds through the plasma membrane [24–26]. However, whether the dipteran ABC transporters are associated with the insecticidal activity of Bt Cry toxins has not been investigated. In this study, we explore the *A. aegypti* genome to identify the members that belong to ABC transporter superfamily. Then, transcriptomic data were analyzed to determine the profile expression of these proteins in the gut of *A. aegypti* larvae. The orthologous proteins to ABCA2, ABCB1 and ABCC2 transporters acting as receptors for Cry toxins in lepidopteran and coleopteran species were subjected to CRISPR-Cas9 KO. Further studies were performed with the generated *A. aegypti* KO populations, indicating that the KO of *Aae*ABCA2, *Aae*ABCB1 and *Aae*ABCC2 does not confers resistance to Cry4Aa, Cry4Ba, Cry11Aa or Cry11Ba toxins. However, detailed binding assays of the four Cry toxins to the brush border microvilli membrane of gut epithelium revealed reduced binding, thus lack of resistance to Cry toxin did not correlate with reduced binding. Finally, characterization of the expression levels of paralogous ABC transporters, revealed their increased expression in the KO populations, suggesting a compensatory mechanism and a redundant effect as putative Cry receptors limiting resistance evolution.

## Materials and methods

### Insect rearing

The *A. aegypti* Cuernavaca strain was established for over 15 years at Instituto de Biotecnología-UNAM facilities. The insects were reared at 25–28°C, 60–80% relative humidity with a photoperiod of 12h:12h (dark: light). During larval development, the insects were maintained in tap water and fed with commercial dog food. Adults were placed in mesh cages (15 x 15 x 15 cm) and fed with a 10% honeybee solution. Adult females were fed with cattle blood contained in a petri dish sealed with a ParafilmM membrane (Sigma-Aldrich).

### Identification and transcriptomic analysis of ABC transporters

To identify the putative ABC transporters in *A. aegypti* we sought all genes that belong to the superfamily of ABC transporters (PFAM: PF00005) in the assembled genome of *A. aegypti* LVP_AGWG (https://vectorbase.org/vectorbase/app). The retrieved protein sequences were subjected for multiple alignment using the Clustal Omega server (https://www.ebi.ac.uk/jdispatcher/msa/clustalo) and a phylogenetic tree was constructed by neighbor-joining with the identified amino acid sequences using the iTOL online tool (https://itol.embl.de). To gain insight into the expression pattern of ABC transporters in the gut of *A. aegypti* larvae we analyzed their mRNA expression based on gut RNAseq transcriptome data that were previously reported [27]. Transcriptomic data from six independent samples of gut tissues from fourth-instar larvae were analyzed to calculate the read counts and the results were normalized in TPM units (transcripts per million of kilobase). Structural domains of ABC transporters were predicted using the EMBL-EBI server (http://www.ebi.ac.uk/Tools/hmmer/search/phmmer).

### Design and synthesis of sgRNAs

To design the sgRNA’s for targeting the ABC transporter genes by CRISPR-Cas9, we use the CRISPOR server (http://crispor.gi.ucsc.edu/). The exons located at 5’ end of *Aae*ABCA2 (*AAE*L012698), *Aae*ABCB1 (*AAE*L008134) and *Aae*ABCC2 (*AAE*L025460) were submitted to the CRISPOR server to search for the 5’-N_20_NGG-3’ motifs. Among the motifs identified, the DNA sequences showing the highest score of gene edition efficiency displayed in the CRISPOR server were selected for targeting each ABC transporter gene: sgRNA-137fw for targeting the second exon of *Aae*ABCA2 (N_20_ = 5’-GCTCATTCTTGTACGAGGAC-3’), sgRNA-235rv for targeting the second exon of *Aae*ABCB1 (N_20_ = 5’-AATCGCACCACGGTCAGTAC-3’) and sgRNA-134rv for targeting the third exon of *Aae*ABCC2 (N_20_ = 5’- AGCAACCAGCCAAGGAAGAT-3’). Synthesis *in vitro* of sgRNAs was carried out by two steps procedure. The first step consists of synthetizing the DNA template by assembly PCR using two oligonucleotides. One specific oligonucleotide containing the T7 promoter sequence at 5’ end, followed by the N_20_ specific sequence for each ABC transporter and at 3’ end of this oligonucleotide, it contains an overlapping sequence for assembly. A second common oligonucleotide containing the tracrRNA sequence was designed (S1 Table). Briefly, 50 pmol of oligonucleotides were assembled by PCR with Phusion DNA-polymerase using the following program cycling: 35 cycles of 98°C for 10 s, 58°C for 10 s and 72°C for 10 s. Finally, the PCR products were purified using DNA Clean and concentrator kit (Zymo Research). For the second step, the PCR products were used as DNA template for *in vitro* transcription to obtain the sgRNA’s using the TranscriptAid T7 High Yield Transcription kit following the protocol described by the manufacturer (Thermo Scientific).

### *Aedes aegypti* eggs microinjection

To collect freshly laid *A. aegypti* eggs, female mosquitoes were blood-fed and, after 2–3 days, oviposition was induced by placing a water container with moist paper in the cage and incubating it in darkness. Eggs for microinjection were collected within 2 h post-oviposition and lined up on moister filter paper upon stereomicroscope. Eggs were carefully transferred to double-sided tape on a microscope glass slide and covered with halocarbon oil 700 (Sigma Aldrich). Needles of aluminosilicate glass were generated with the P-97 Micropipette puller (Sutter Instrument) with the following setting parameters, heat = 548, pull = 60, velocity = 60, delay = 90, pressure = 300. Needle tip was then beveled with the MicroGrinder EG401 (Narishige). Approximately 0.5 nl of a mixture containing 300 ng/µl of Cas9 nuclease NLS (Dharmacon Reagents) and 250 ng/µl of sgRNA were injected at posterior side of eggs using the Micromanipulator MM3301-R (World Precision Instrument) and the Pneumatic PicoPump PV830 (World Precision Instrument). Once the eggs were injected, the oil was removed carefully with clean paper, and eggs were maintained in a humidity chamber for 4 days. Afterward, the eggs were submerged in ddH_2_O for larvae hatching.

### Genotypification

Single couples were mated and once females laid eggs, the genomic DNA (gDNA) was isolated from the whole body of adult insects with the Quick-DNA Miniprep Plus kit (Zymo Research). Subsequently, the gDNA was used as template for PCR to amplify a gene fragment of ABC transporters containing the sgRNA hybridization site (S1 Table). The INDELs generated by CRISPR-Cas9 were identified by *in vitro* Cas9 digestion. Briefly, 200 ng of PCR fragments were cleaved with the complex Cas9:sgRNA (30:25 ng/µl) in a reaction supplemented with Buffer3.1 (NewEngland BioLabs) during 1 h at 37°C. The reaction was then analyzed by electrophoresis in 1% agarose gel. Samples containing the DNA fragment that remained uncleaved or exhibited partial cleavage were chosen for DNA sequencing to confirm the presence of INDELs.

### Quantitative RT-qPCR

Total RNA was isolated from gut tissue dissected of fourth-instar larvae of *A. aegypti* with Quick-RNA Miniprep (ZymoResearch). The cDNA was synthesized with an Oligo (dT) 20-mer Primer from 2 μg of total RNA using the SuperScript III reverse transcriptase kit (Invitrogen Life Technologies). The ribosomal protein S3 (*rpS3*) gene was used as housekeeping control. Oligonucleotides for RT-qPCR are listed in S1 Table. Quantitative RT-qPCR assays were performed using Maxima SYBR Green/ROX qPCR Master Mix (Thermo Scientific) in a 48-well plate according to the manufacturer’s instructions. Reactions were performed using an Eco real-time PCR system (Illumina). Melting curve analysis and relative quantification (2^ΔΔ^CT method) were performed using EcoStudy software (Illumina).

### Production and labeling of Cry toxins

The Bt crystal inclusions of Cry11Aa, Cry11Ba, Cry4Aa and Cry4Ba toxins were produced in the different Bt strains following the procedure described previously [10]. Crystals were purified by ultracentrifugation in a discontinuous sucrose gradient and labeled with Alexa Fluor 567 as was described previously [5].

### Confocal microscopy

Three to six *A. aegypti* fourth-instar larvae were placed into 24-wells plate containing 2 ml of dH_2_O per well. Inclusion crystals of Cry11Aa, Cry11Ba, Cry4Aa or Cry4Ba toxins previously labeled with Alexa Fluor 567 were added at 1 µg/ml final concentration and larvae were incubated for 3 h in darkness. Subsequently, gut tissues were dissected and fixed overnight (12 h) in a fixing solution (4% paraformaldehyde, 5% sucrose, PBS pH 7.4) at room temperature. Next, the fixed tissues were rinsed three times with PBS, cell nuclei were stained in a solution of 5 mg/ml of 4’,6-diamidino-2-fenilindol (DAPI)/PBS for 30 min and rinsed again three times with PBS. Finally, the gut tissues were placed on microscope glass-slides and covered with Prolong Glass antifade mountant (Thermo Fisher Scientific). Gut tissues were examined using confocal laser scanning microscope (Olympus FV1000) at the Laboratorio Nacional de Microscopía Avanzada of the Instituto de Biotecnología-UNAM facilities. Images were collected with a 20X or oil immersion 60X S objective (numerical aperture: 1.3).

### Bioassays

To estimate the concentration of Cry toxin that kill 50% of *A. aegypti* larvae (LC_50_) bioassays were carried out by suspending the mixture spore/crystal in 100 ml of dH_2_O containing 10 fourth-instar larvae. Mortality was registered after 24 h and LC_50_ values were estimated by Probit analysis using the PoloPlus software (LeOra Software Company).

## Results

### Phylogenetic classification of *A. aegypti* *ABC* transporters

**Fig 1:**
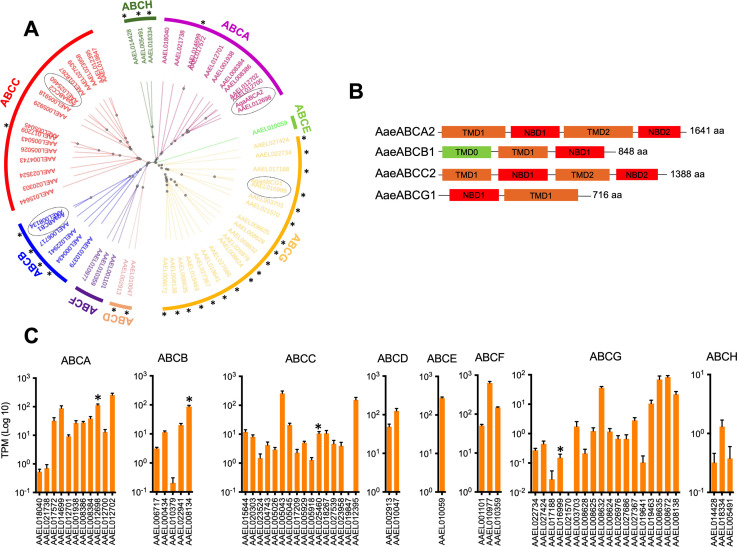
The *A. aegypti* ABC transporters. **(A)** Phylogenetic tree of *A. aegypti* ABC transporters based on their aminoacid sequences. The eight ABCA-H protein subfamilies are grouped in clades showed with different colors. The ABCA2, ABCB1, ABCC2 and ABCG1 of *Anopheles gambiae* and their orthologous proteins in *A. aegypti* are enclosed in dashed lines. Half-transporters are marked with an asterisk. **(B)** Structural protein organization of *Aae*ABCA2, *Aae*ABCB1, *Aae*ABCC2 and *Aae*ABCG1. NBD: Intracellular nucleotide-binding domain, TMD: Transmembrane domain. **(C)** Analysis of gene expression of *A. aegypti* ABC transporters in the gut tissue of forth-instar larvae. The expression level was normalized to transcripts per million (TPM) for all small RNAs in the sequenced sample. Bars marked with an asterisk correspond to *Aae*ABCA2, *Aae*ABCB1, *Aae*ABCC2 and *Aae*ABCG1.

A total of 59 putative ABC transporters were found in the *A. aegypti* genome, among them 30 are full transporters since they contain two cytosolic nucleotide-binding domains (NBD), while 29 are half transporter with a unique NBD. The phylogenetic tree of the 59 proteins showed eight clades that are consistent with the different ABC transporter subfamilies (Fig 1A). The three largest subfamilies are composed of 11, 16 and 18 members that belong to ABCA, ABCC and ABCG subfamilies, respectively. The rest of the clades correspond to the ABCB subfamily with 5 members; ABCF and ABCH with 3 members each; ABCD with 2 members; and a unique member belongs to ABCE subfamily.

Since the annotation of *A. aegypti* genes is not well-defined, we compared the known ABC transporters from *Anopheles* gambiae to identify the orthologous proteins to ABCA2, ABCB1, ABCC2 and ABCG1, which are recognized as Cry toxin receptors in a certain number of lepidopteran or coleopteran insect species. The amino acid sequences of the orthologous ABC transporters from *A. gambiae* were included in the phylogenetic analysis and their closest members were considered as orthologous proteins in *A. aegypti* (Fig 1A). The *AAE*L012698 was the member from the ABCA subfamily that showed the highest homology to AgABCA2 (AGAP006380) showing 64.05% identity. This protein shows the typical structure of full ABC transporters, composed of two NDB and two alfa-helical transmembrane domains (TMD) (Fig 1B). The *AAE*L008134 was closely related to AgABCB1 (AGAP002278) showing 80.47% identity. The *AAE*L008134 is a half transporter since it contains a single NBD and TMD, but it also contains an extra N-terminal transmembrane domain (TMD0), which is typical of ABCB members. The full transporter *AAE*L025460 was closely related to AgABCC2 (AGAP001775) showing 76.65% identity. The orthologous protein to AgABCG1 (AGAP000553) was the *AAE*L016999 with 80.33% identity, showing a structure of half transporter. For more clarity, hereafter these *A. aegypti* ABC transporters are named as *Aae*ABCA2 (*AAE*L012698), *Aae*ABCB1 (*AAE*L008134), *Aae*ABCC2 (*AAE*L025460) and *Aae*ABCG1 (*AAE*L016999).

### Profile expression of *ABC* transporters in the gut of *A. aegypti* larvae

Based on gut epithelium RNAseq transcriptome data that were previously reported [27], among the 11 members from ABCA subfamily, the *Aae*ABCA2 and *AAE*L014699 transporters showed a relatively high expression with mean TPM values of 113 and 87, respectively (Fig 1C). Interestingly, the *AAE*L012702 gene presented the highest expression level, while its closest related *AAE*L012700 gene is one of members with the lowest expression from this subfamily. These latter two ABCA transporters, which seem being product from gene duplication, are also closely related to *Aae*ABCA2 transporter. The *AAE*L018040 and *AAE*L021738 genes did not show expression in the larvae gut. Regarding the gene expression of ABCB subfamily, the *Aae*ABCB1 half transporter exhibited the higher expression levels of this subfamily with a mean TPM value of 87, the *AAE*L006717 is not expressed, and the other three members showed low expression. In the case of ABCC transporters, most of the genes showed low expression, with TPM values that ranged from 1.2 to 12, including the *Aae*ABCC2 transporter (10.5 TPM). Among 16 members from ABCC transporters subfamily, only two genes, *AAE*L005043 and *AAE*L012395, are highly expressed. Similar to the ABCA subfamily organization, two closely related ABCC transporters, the *AAE*L012395 and *AAE*L019847, were identified. The *AAE*L012395 gene from the ABCC subfamily showed one of the highest expression levels of this subfamily while the *AAE*L019847 did not show expression. Our results also indicated that most of the ABCG’s transporters showed low expression. The *Aae*ABCG1 transporter is marginally expressed with a TPM value of 0.15. Members of ABCD, ABCE and ABCF subfamilies are the ABC transporters that showed the higher expression, particularly the *AAE*L010977 of the ABCF subfamily which is the ABC transporter highly expressed among the 59 ABC transporters of *A. aegypti* with a mean TPM value of 628. On the other hand, the three members of ABCH subfamily exhibited reduced or minimal expression with TPM values <1.5.

To validate the presence of transcripts for *Aae*ABCA2, *Aae*ABCB1, *Aae*ABCC2 and *Aae*ABCG1 we carried out end-point RT-PCR. The RT-PCR results showed a transcript abundance consistent with the RNA-seq data, following the order: *AaeABCB1* > *AaeABCA2* > *AaeABCC2* > *AaeABCG1* (S1 Fig).

### *CRISPR*-Cas9 knockout of *AaeABCA*2, *AaeABCB*1 and *AaeABCC*2

**Fig 2:**
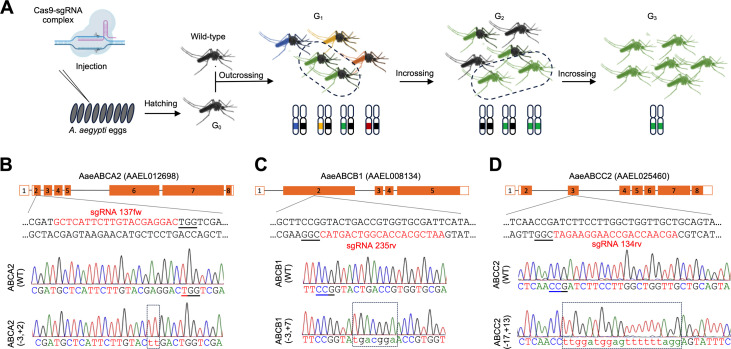
CRISPR-Cas9 knockout of *A. aegypti* ABC transporters. **(A)** Schematic representation of the strategy to generate the *A. aegypti* homozygous KO-mutants. Dashed lines ovals indicate the insect couple with the same haplotype mutation that were selected for crossing to obtain the generation 1 (G_1_), 2 (G_2_) and 3 (G_3_). **(B)** CRISPR-Cas9 KO of *Aae*ABCA2, **(C)** *Aae*ABCB1 and [50] *Aae*ABCC2. The upper panels show the genomic structure of *Aae*ABCA2, *Aae*ABCA2 and *Aae*ABCA2 genes. CDS and UTR are showed in orange and white rectangles, respectively. The sgRNA sequence for CRISPR-Cas9 targeting the exons are also showed. The bottom panels show the DNA-sequencing chromatogram of wild-type and homozygous mutant alleles, indicating the INDELs in dashed line rectangles with mutated bases in lower case. PAM sequences are underlined.

Currently, there is no-direct evidence of the role of ABC transporters in the mode of action of Cry toxins in dipteran species. We decided to begin our work with a set of candidates based on conserved mechanisms as Cry toxin receptors in other insect orders. We used the CRISPR-Cas9 system to generate the KO mutants of *Aae*ABCA2, *Aae*ABCB1 and *Aae*ABCC2 transporters in *A. aegypti*. Despite that ABCG1 transporter have also been related with Cry toxin resistance, here we excluded the *Aae*ABCG1 transporter because the analysis of transcriptomic data showed marginal expression in gut epithelial cells (Fig 1C and S1 Fig). To generate the KO mutant of each ABC transporter we co-injected the endonuclease Cas9 and a specific sgRNA into 200 pre-blastoderm eggs of *A. aegypti* for targeting the corresponding exon region (Fig 2A). Approximately 5% of injected eggs hatched and larvae were developed to adulthood (G_0_). G_0_ adults were outcrossed with *A. aegypti* wild-type strain, allowing the isolation of germ-line mutations. Once females laid eggs to obtain the first generation (G_1_), the G0 insects were analyzed to detect INDELs by digestion of the PCR product with Cas9-sgRNA complex and subsequent DNA sequencing. The offspring of G_1_ that presented mutation were incrossed in single couples obtaining G_2_. The couples that presented identical mutations, were selected to obtain the homozygous population (Fig 2A).

Mutations that generated a frameshift in the sequence that produce premature stop codons on the predicted exons were selected. The second exon of *Aae*ABCA2 transporter was targeted with the sgRNA-137fw generating an INDEL of a 3-bp deletion and a 2-bp insertion (Fig 2B). The *Aae*ABCB1 transporter was also targeted at second exon with the sgRNA-235rv, which presented an INDEL of a 3-bp deletion and a 7-bp insertion (Fig 2C). The *Aae*ABCC2 transporter was targeted at third exon using the sgRNA-134rv generating an INDEL of a 17-bp deletion and a 13-bp insertion (Fig 2D).

### Response of genetically modified *A. aegypti* to Cry toxins

To evaluate the effect of *Aae*ABCA2-KO, *Aae*ABCB1-KO and *Aae*ABCC2-KO on the *A. aegypti* susceptibility to four mosquitocidal Cry toxins, we carried out a preliminary bioassay with F1 homozygous insects using a single dose corresponding to 2-fold LC_50_ value of Cry4Aa, Cry4Ba, Cry11Aa and Cry11Ba toxins. These assays showed that after 24 h of Cry toxin treatment, the Cry4Aa and Cry11Aa failed to kill *Aae*ABCA2-KO larvae, while Cry4Ba and Cry11Ba were ineffective against *Aae*ABCB1-KO larvae. However, 100% of mortality was observed after 48 h (S2 Fig). Subsequently, F1 homozygous insects were incrossed in mass to obtain the KO populations and fourth-instar larvae were exposed during 24 h to different concentrations of Cry4Aa, Cry4Ba, Cry11Aa and Cry11Ba to estimate the LC_50_. However, no significant changes of Cry toxin insecticidal activity were observed in these bioassays, since their LC_50_ and 95% fiducial limits values were comparable to *A. aegypti* wild-type (Table 1).

**Table 1:** Susceptibility of *A. aegypti* populations to mosquitocidal Cry toxins. Values are given in ng of Cry toxin/ml and 95% fiducial limits are showed in parenthesis.

	Cry11Aa	Cry11Ba	Cry4Aa	Cry4Ba
***Aae*Wild-type**	198.6 (133.0-292.5)	86.1 (38.1-127.9)	1121 (292.2-1679.5)	180.3 (105.8-263.0)
***Aae*ABCA2 KO**	123.6 (40.3-213.6)	53.0 (29.9-80.9)	1271.0 (687.9-2111.3)	110.0 (61.6-161.5)
***Aae*ABCB1 KO**	217.9 (134.0-336.6)	58.4 (22.7-85.9)	985.7 (703.4-1317.4)	181.8 (129.3-248.3)
***Aae*ABCC2 KO**	127.8 (92.7-169.7)	76.3 (50.4-102.0)	959.4 (700.5-1248.6)	113.8 (74.9-152.3)

### Binding of Cry toxin to microvilli of *A. aegypti* gut cells

**Fig 3:**
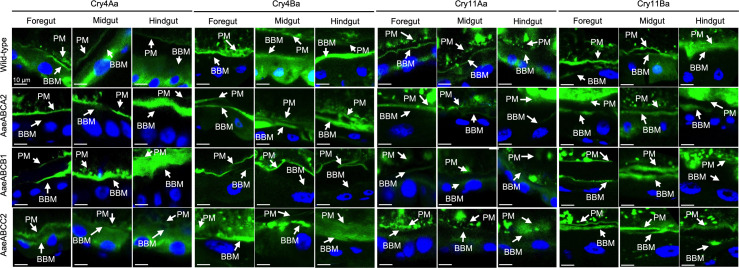
Histological analysis of *A. aegypti* gut tissues. Micrographs of confocal microscopy showing the binding of Cry toxins labeled with AlexaFluor 567 (showed green) to peritrophic membrane (PM) or brush border membrane (BBM) of *A. aegypti* larvae gut, either wild-type or CRISPR-Cas9 KO populations of ABCA2, ABCB1 and ABCC2. Nucleus were stained with DAPI (showed blue). Scale bars are 10 μm.

*In vivo* binding assays were conducted to analyze whether ABCA2, ABCB1 and ABCC2 serve as receptors for mosquitocidal Cry toxins. *A. aegypti* four-instar larvae were fed with fluorescent labeled crystals for 3 h. Gut tissues were subsequently visualized using confocal microscopy (Fig 3). In wild-type larvae, all four Cry toxins bound to the peritrophic membrane (PM) and brush border microvilli (BBM) through the whole gut. In addition, the micrographs showed that Cry toxins were internalized into certain epithelial cells. This phenomenon was previously described and it is indicative of epithelial tissue damage caused by Cry toxin activity [5,28]. In *A. aegypti* larvae with ABC transporter KO, binding of the four Cry toxins was observed on the PM. Notably, binding of some Cry toxins to the BBM was significantly reduced in certain ABC-KO strains. For instance, Cry4Aa and Cry11Aa exhibited significant decreased binding to the BBM in the *Aae*ABCA2-KO, whereas Cry4Ba showed reduced binding to the BBM of *Aae*ABCB1-KO. Similarly, Cry11Ba showed reduced binding exclusively in the hindgut region of the *Aae*ABCB1-KO. These data correlate with the preliminary bioassay showing reduced toxicity of the F1 insects. Interestingly, in these knockout tissues, toxin internalization into epithelial cells was also reduced, with exception of *Aae*ABCC2-KO midgut samples, where Cry4Aa and Cry11Aa toxins were clearly detected inside the cells. To quantify the Cry toxins bound to the BBM, a radiometric analysis was performed, comparing fluorescence line-intensity between the PM and BBM. The analysis revealed a significant reduction in Cry toxin binding to the BBM (S3 Fig).

### Transcription of paralogous ABC transporter genes is upregulated in response to the KO of their corresponding ABC transporter genes

**Fig 4:**
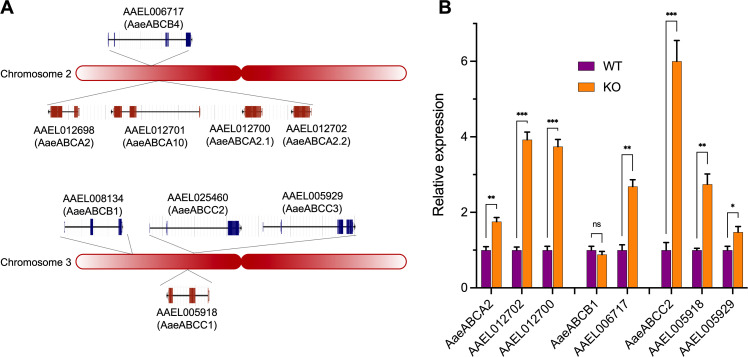
Quantitative analysis of ABC transporters transcripts. **(A)** Distribution of *Aae*ABCA2, *Aae*ABCB1, *Aae*ABCC2 and their paralogous genes in the *A. aegypti* chromosomes. **(B)** The expression level of paralogous genes of *Aae*ABCA2, *Aae*ABCB1, and *Aae*ABCC2 transporters was analyzed by RT-qPCR and compared with the *A. aegypti* wild-type (WT) and ABC-KO (KO) populations. Error bars represent the SEMs from triplicate assays. Multiple unpaired t-test was performed to obtain the *P* values. Asterisks indicate statistically significant differences: *P* ≤ 0.05 (*), *P* ≤ 0.01 (**), *P* ≤ 0.001 (***), ns: not significant (*P* ≥ 0.05).

Since binding and LC_50_ values of KO mutants did not correlate, we analyzed the expression levels of phylogenetically related genes to *Aae*ABCA2, *Aae*ABCB1, and *Aae*ABCC2 by RT-qPCR in the gut tissue (Fig 4). Transcript levels of *AAE*L012702 (*Aae*ABCA2.1) and *AAE*L012700 (*Aae*ABCA2.2), which are paralogs of *Aae*ABCA2, revealed that both genes were 4-fold overexpressed in the *Aae*ABCA2 KO compared to the wild-type strain. Similar results were observed for *AAE*L006717 (*Aae*ABCB4), which is phylogenetically related to *Aae*ABCB1, showing 3-fold increased expression in the *Aae*ABCB1 KO strain compared to the *A. aegypti* wild-type population. Additionally, *AAE*L005918 (*Aae*ABCC1) and *AAE*L005929 (*Aae*ABCC3), which are phylogenetically related to *Aae*ABCC2, were also upregulated in the *Aae*ABCC2 KO strain than in wild type strain. It is worth noting that most of paralogous genes showed low expression in the transcriptomic analysis with TPM values <10 (Fig 1). These results suggests that ABC transporters KO in *A. aegypti* was compensated by upregulation of paralogous genes.

## Discussion

Identification of Bt Cry toxins receptors is fundamental for understanding their specificity against insect targets and the resistance mechanisms developed by insect pests. The pore-forming mechanism of these toxins proposes that membrane proteins such as cadherin-like proteins, aminopeptidases and alkaline phosphatases bind to Cry toxins and modulate the insecticidal activity. However, CRISPR-Cas9 KO of these proteins in *A. aegypti* did not significantly change the susceptibility to Cry toxins [9,10], suggesting that additional proteins may also play a role as Cry receptors. The ABC transporters, a heterogeneous family of transmembrane proteins, have also been shown to have an important role assisting Cry toxin activity in lepidopteran and coleopteran species [29].

We identified 59 ABC transporter proteins containing NBD domains in the *A. aegypti* genome, which were grouped into 8 subfamilies according their aminoacid sequence identity. This analysis is consistent with the content of ABC transporters from arthropods [24]. Based on RNAseq data, their expression profile in *A. aegypti* gut tissue shows the presence of ABCA2, ABCB1 and ABCC2. Since it was demonstrated that orthologous of ABC transporter proteins serve as receptors for Cry toxins among different insect species, we hypothesized that dipteran ABC transporters could also serve as Cry receptors. Among the 59 ABC transporters identified in *A. aegypti*, we selected *Aae*ABCA2, *Aae*ABCB1, and *Aae*ABCC2 for initial analysis as putative Cry toxin receptors. Although *A. aegypti* is a dipteran species and not closely related to Lepidoptera or Coleoptera, where the role of ABC transporters in Cry toxin activity is well-characterized, we acknowledge that functional divergence may have occurred. Consequently, Cry toxicity in *A. aegypti* could involve other ABC transporters or entirely different membrane proteins. Nevertheless, due to the lack of direct evidence regarding the involvement of ABC transporters in the Cry toxin mode of action in dipteran species, we chose to begin with a focused set of candidates based on conserved mechanisms identified in other insect orders. Therefore, we used the CRISPR-Cas9 system to generate KO mutants of the ABCA2, ABCB1, and ABCC2 orthologous proteins in *A. aegypti* to gain insight into their role in the mode of action of Cry toxins. In contrast to the KO mutations of ABCA2 and ABCC2 in lepidopteran species, the KO of *Aae*ABCA2 and *Aae*ABCC2 did not alter the toxicity of Cry4Aa, Cry4Ba, Cry11Aa and Cry11Ba in *A. aegypti*. Selection of naturally occurring mutations in the ABCA2 transporter in the field or laboratory selected lepidopteran populations such as *P. gossypiella*, *H. armigera* and *H. punctigera* have been linked to resistance against the Cry2Ab toxin [20,21]. This ABCA2-linked resistance phenotype has been validated by genome edition in at least five different lepidopteran species, such as *H. armigera*, *H. zae*, *T. ni*, *B. mori*, and *P. gossypiella* [30–34]. Similarly, resistant phenotype to Cry1A/Cry1F toxins in lab/field-selected insect such as *H. armigera*, *S. exigua*, *S. frugiperda*, *T. ni*, *P. xylostella* and *B. mori*, linked to the ABCC2 transporter [12,35–38] were validated by KO mutations [15,41,42]. However, the resistance levels to Cry1A/Cry1F toxins are low in these edited ABCC2 KO mutated insects [15,41,42]. Thus, it was proposed a redundant role of ABCC2 and ABCC3 transporters, since double KO of these two paralogous proteins confer significantly higher levels of Cry toxin resistance than single KO of either ABCC2 or ABCC3 in *H. armigera* or *P. xylostella* [15,39,40]. This redundant effect was not observed when the paralogous ABCA1 and ABCA2 were analyzed in *H. armigera* [21].

In the case of *Aae*ABCB1 KO, we also did not observe a resistance phenotype to the mosquitocidal Cry4Aa, Cry4Ba, Cry11Aa and Cry11Ba toxins. Initially, ABCB1 was identified as putative receptor for Cry3Aa toxin in the coleopteran *Chrysomela tremuela* [22]. The homologous ABCB1 protein of *Diabrotica virgifera virgifera* was transiently expressed in HEK293T cells rendering susceptibility to Cry3A toxin, whereas knockdown expression by RNAi of *Dv*ABCB1 in *D. virgifera virgifera* larvae, resulted in higher tolerance to Cry3A toxin. However, the resistance associated to ABCB1 was not confirmed by KO mutations induced by CRISPR-Cas9 in any coleopteran specie. Recent studies highlight its role as putative receptor for Cry1Ac toxin in lepidopteran, since reduced gene expression of ABCB1 showed significant resistance to Cry1Ac toxin in *P. xylostella* larvae [41]. Further studies revealed that CRISPR-Cas9 ABCB1 KO in *B. mori* confers resistance to Cry1Ia, Cry1Ba and Cry9Da toxins, and that transiently expression of *Px*ABCB1 or *Bm*ABCB1 in HEK293T cells induce susceptibility to Cry1Ba or Cry1Ia and Cry9Da, respectively [42]. The susceptibility to Cry1Aa, Cry1Ab, Cry1Ac, Cry1Ca, Cry1Da, Cry1Fa, Cry2Ab and Cry9Aa, of BmABCB1 KO larvae was analyzed, but no resistance was observed. These results correlate with CRISPR-Cas9 ABCB1 KO mutation in *S. frugiperda*, where also no resistance was observed against Cry1Ab and Cry1Fa toxins [43]. However, transient expression of *Sf*ABCB1 in Sf9 cells render susceptibility to the chimeric Cry1B.868 toxin [44], which is consistent with the hypothesis that *Bm*ABCB1 is a receptor for Cry1B toxin family. Interestingly, *Sf*ABCB1 KO increased the susceptibility of *S. frugiperda* to chemical pesticides [43]. Similarly, ABC transporters are also related to chemical pesticide resistance in dipteran [24–26].

The toxin-receptor interaction is an important step in the mode of action of Cry toxins, rendering specificity against the insect target and triggering conformational changes required for oligomerization and membrane insertion. For instance, heterologous expression of *S. frugiperda* or *B. mori* ABCC2 gene in Sf9 cell line conferred susceptibility to Cry1A and Cry1F toxins, while mutation in extracellular loops of ABCC2 reduced Cry toxin binding and susceptibility [45–47]. On the other hand, *Bm*ABCC2 expression in non-sensitive HEK293T cell line also conferred susceptibility to the lepidopteran-specific Cry1Aa toxin, while expression of *Tribolium castaneum* ABCB4 conferred susceptibility to the coleopteran-specific Cry8Ca toxin [48]. These studies demonstrate that the heterologous expression of receptors in cell lines confers susceptibility through the acquisition of a binding component. However, the Cry toxin binding not always directly correlate with toxicity, since SPR analysis showed an interaction between Cry3Bb with *Tc*ABCB4, but expression of *Tc*ABCB4 in HEK293T cells did not confer susceptibility to Cry3Bb toxin [48]. Similar results were reported with Cry4Aa, Cry11Aa and Cry11Ba toxins in *Culex pipiens*, since their binding did not directly correlate with toxicity [49]. Interestingly, we found that the selected *Aae*ABCA2-KO, *Aae*ABCB1-KO, and *Aae*ABCC2-KO populations did not show altered susceptibility to Cry4Aa, Cry4Ba, Cry11Aa, or Cry11Ba toxins. However, *in vivo* binding analyses revealed significantly reduced binding of Cry4Aa and Cry11Aa toxins to BBM from *Aae*ABCA2-KO larvae, and reduced binding of Cry4Ba and Cry11Ba to BBM from *Aae*ABCB1-KO larvae. The role of *Aae*ABCA2 or *Aae*ABCB1 as receptors for Cry4Aa/Cry11Aa or Cry4Ba/Cry11Ba, respectively could be confirmed by the future expression of these ABC transporters in insect cell line.

As mentioned above, we hypothesized that paralogous of ABC transporters might play a crucial role in compensating the KO of *Aae*ABCA2, *Aae*ABCB1, and *Aae*ABCC2. This phenomenon of genetic compensation by loss-of-function has been analyzed exhaustively [50,51]. For instance, the lepidopteran *T. ni* populations that have developed resistance to Cry1Ac toxin, revealed that the resistance was linked to downregulation of the APN1 receptor. Interestingly, the APN6 isoform in this strain was upregulated to compensate the loss of APN1 [52]. As was mentioned above, functional redundance of ABCC2 and ABCC3 as Cry toxin receptors was observed in several lepidopteran species. However, these reports did not analyze the genetic compensation by up/downregulation of related paralogous genes. It is reasonable to consider that a compensation mechanism might also be occurring, as the CRISPR-Cas9 KO of ABCC2 does not correlate with the high levels of resistance observed in the laboratory/field selected mutations of this protein. Our phylogenetic analysis of the ABC transporter repertoire in *A. aegypti* genome, allowed us to identify putative paralogous genes of *Aae*ABCA2, *Aae*ABCB1 and *Aae*ABCC2 genes. Expression analysis of these paralogous gene revealed upregulation in the corresponding ABC KO strains, which is consistent with genetic compensation mechanism in response to the KO of these ABC transporters. A possible explanation why the KO mutations in *Aae*ABCA2, *Aae*ABCB1 or *Aae*ABCC2 did not alter the susceptibility to Cry toxins in *A. aegypti*, could involve this compensation in paralogous expression that display redundant function. To further explore this possibility, future experiments should consider combinatorial approaches, such as double or triple KO, including not only *Aae*ABCA2, *Aae*ABCB1 and *Aae*ABCC2 but also their paralogous proteins, or other known Cry- binding proteins like Cadherin, mALP or APNs.

In summary, the CRISPR-Cas9 KO of *Aae*ABCA2, *Aae*ABCB1 and *Aae*ABCC2 did not confer resistance to Cry4Aa, Cry4Ba and Cry11Aa toxins from Bti or Cry11Ba toxin from Bt subsp. jegathesan. However, the binding of some Cry toxins to the gut microvilli was reduced significantly in some of the KO mutants of *A. aegypti*. These three *A. aegypti* ABC transporters are expressed in the larval midgut epithelium and are phylogenetically related to orthologous proteins linked to Cry toxin resistance phenotypes previously described in lepidopteran and coleopteran species. Our result suggest that the resistance phenotype may be masked by compensatory upregulation of paralogous ABC transporters, which could functionally substitute the disrupted genes and maintain susceptibility to the Cry toxins. The potential redundant functional role as Cry toxin receptors of these paralogous proteins remains unknown and require further analysis in *A. aegypti*.

## Supporting information

S1 Table List of oligonucleotides for sgRNA synthesis, PCR and qPCR. (DOCX)

S1 Fig Analysis of ABC transporters transcripts from *A. aegypti.* Total mRNA from fourth-instar larvae of *A. aegypti* wild-type was isolated and cDNA was synthetized using an oligo-dT. An end-point RT-PCR was performed using specific oligonucleotides to amplify the transcript of *Aae*ABCA2, *Aae*ABCB1, *Aae*ABCC2 and ABCG1. The rpS3 gene was included as positive house-keeping control.
(TIFF)

S2 Fig Bioassay of *A. aegypti* populations. Bioassay were performed using 2-folds the LC_50_ of Cry toxins and mortality was registered after 24 h. Bars represent the percentage of mortality of two independent assays.
(TIFF)

S3 Fig Binding analysis of Cry toxins to gut tissues. Radiometric analysis of 20–30 lines with a length 5 μm selected from anterior, medium and posterior regions of *A. aegypti* gut. Fiji software was used to perform a line analysis of fluorescence intensity (IF) of the brush border membrane (BBM) and peritrophic membrane (PM).
(TIFF)
