# Supplementary material for: ABC transporters knockout in Aedes aegypti induces upregulation of paralogous genes, avoiding resistance development to Bacillus thuringiensis Cry toxins
Source: PLoS One. 2025 Jul 3;20(7):e0327221. doi: 10.1371/journal.pone.0327221 (PMC12225802; doi:10.1371/journal.pone.0327221)
Supplement: S1 Table — (DOCX) [file pone.0327221.s001.docx]

**S1 table.** List of oligonucleotides for sgRNA synthesis, PCR and qPCR.

| Purpose | Amplicon | Primer | Sequence (5’🡪3’) |
| --- | --- | --- | --- |
| Gene edition |  | sgRNA 137fw | GAAATTAATACGACTCACTATA**GCTCATTCTTGTACGAGGAC**GTTTTAGAGCTAGAAATAGCAAG |
|  |  | sgRNA 235rv | GAAATTAATACGACTCACTATA**GAATCGCACCACGGTCAGTAC**GTTTTAGAGCTAGAAATAGCAAG |
|  |  | sgRNA 134rv | GAAATTAATACGACTCACTATA**GAGCAACCAGCCAAGGAAGAT**GTTTTAGAGCTAGAAATAGCAAG |
|  |  | T7 common | AAAAGCACCGACTCGGTGCCACTTTTTCAAGTTGATAACGGACTAGCCTTATTTTAACTTGCTATTTCTAGCTCTAAAAC |
| PCR | ABCA2 (911 bp) | ABCA2-F | GCCGCTTCCTCATGAATG |
|  |  | ABCA2-R2 | GGCCTTCCAGGTCACGAGAG |
|  | ABCB1 (953 bp) | ABCB1-F2 | CTTTGTTTGGTTTAATCTCGTTCTG |
|  |  | ABCB1-R2 | GATTTTCTTGTTGTAGATCGGAACG |
|  | ABCC2 (770 bp) | ABCC2-F2 | GTTCATTCGATGACATTCTAATGAC |
|  |  | ABCC2-R2 | GAATGCAGGAAAACTGATACAATG |
| qPCR | Rsp3 (227 bp) | Rps3Ae Fwd | GGCATGTTCCGTGCTGAATTGAACG |
|  |  | Rps3Ae Rev | TTCTCGGCGTACAGCTCGACG |
|  | ABCA2 (147 bp) | qPCR A2 F (+ ABCA2-R2) | CTACGCCTCCGGTTATTCCC |
|  | ABCA2.1 (181 bp) | A2.1 F | GCTGGATACTCAAGTGATCGC |
|  |  | qPCR A2.1 R | GAGCAGACCAAAACCGAACC |
|  | ABCA2.2 (179 bp) | A2.2 F | CTGAACATCTCAACCATTAGCTTC |
|  |  | qPCR A2.2 R | ATCAGTCCACCAAATACGCG |
|  | ABCB1 (117 bp) | qPCR B1 F (+ ABCB1-R2) | CAGGATCAGGACAACCAATCC |
|  | ABCB4 (156 bp) | qPCR B4 F | CAGTGCTGATATCGCTGAGC |
|  |  | qPCR B4 R | ATCCCAACAGAACGGCAATC |
|  | ABCC2 (167 bp) | qPCR C2 F (+ ABCC2-R2) | AGCTTTCAGAACGGGATGAAGG |
|  | ABCC1 (166 bp) | qPCR C1 F | GTGGACTGTACATGACATTCGG |
|  |  | qPCR C1 R | CCCCTTACAAACAAGCCAGAC |
|  | ABCC3 (162 bp) | qPCR C3 F | GGATGAGAATTGCTTGCTGC |
|  |  | C3 R | GCAACACCCAGACGTAATG |
|  | ABCG1 (159 bp) | qPCR G1 F | CCGGTAAAACAACCTTGCTG |
|  |  | qPCR G1 R | CGGGAATGAAAAGATCGTCC |
