# Supplementary figures and images for: ABC transporters knockout in Aedes aegypti induces upregulation of paralogous genes, avoiding resistance development to Bacillus thuringiensis Cry toxins
Source: PLoS One. 2025 Jul 3;20(7):e0327221. doi: 10.1371/journal.pone.0327221 (PMC12225802; doi:10.1371/journal.pone.0327221)

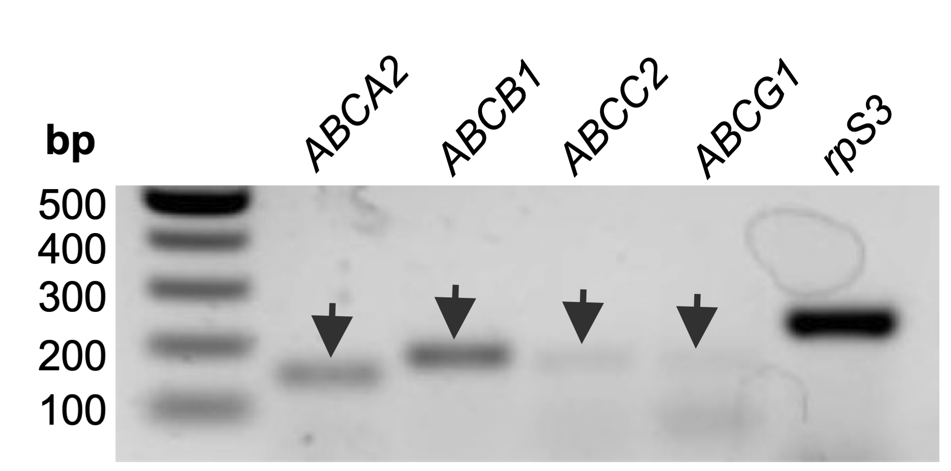

Supplement: S1 Fig — Total mRNA from fourth-instar larvae of A. aegypti wild-type was isolated and cDNA was synthetized using an oligo-dT. An end-point RT-PCR was performed using specific oligonucleotides to amplify the transcript of AaeABCA2, AaeABCB1, AaeABCC2 and ABCG1. The rpS3 gene was included as positive house-keeping control. (TIFF) [file pone.0327221.s002.tiff]

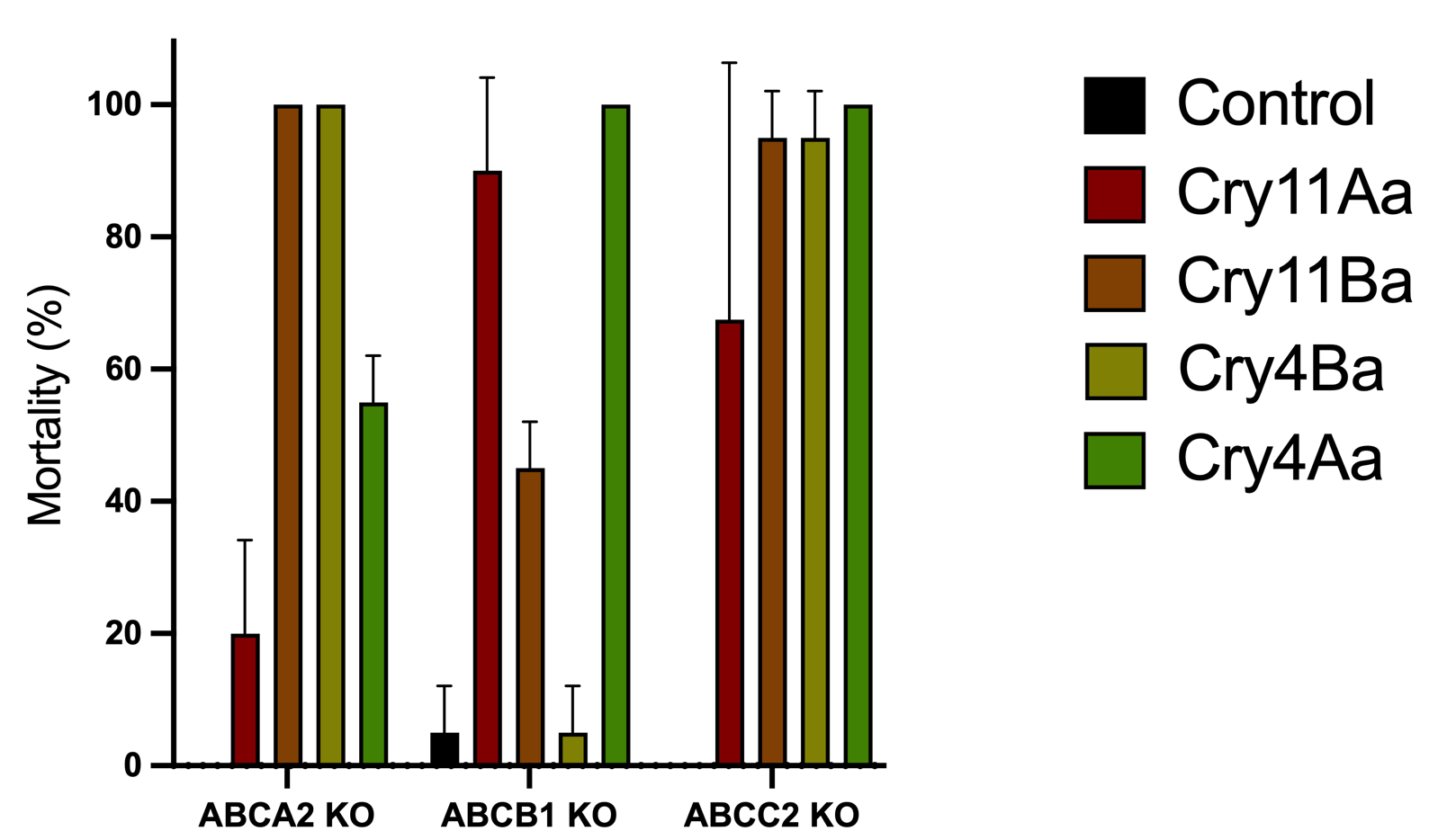

Supplement: S2 Fig — Bioassay were performed using 2-folds the LC50 of Cry toxins and mortality was registered after 24 h. Bars represent the percentage of mortality of two independent assays. (TIFF) [file pone.0327221.s003.tiff]

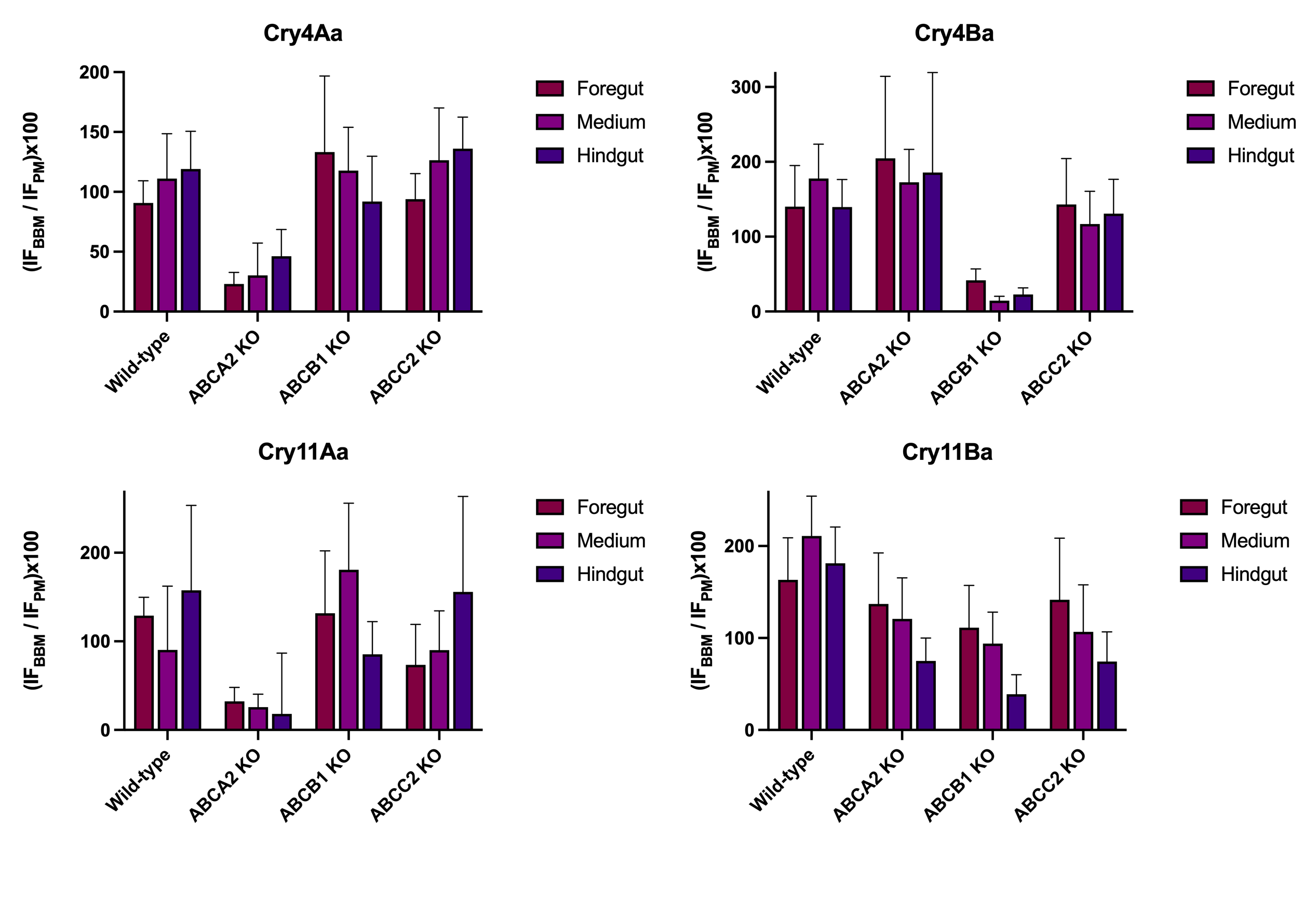

Supplement: S3 Fig — Radiometric analysis of 20–30 lines with a length 5 μm selected from anterior, medium and posterior regions of A. aegypti gut. Fiji software was used to perform a line analysis of fluorescence intensity (IF) of the brush border membrane (BBM) and peritrophic membrane (PM). (TIFF) [file pone.0327221.s004.tiff]
